# Phenethyl Isothiocyanate Suppresses the Proinflammatory Cytokines in Human Glioblastoma Cells through the PI3K/Akt/NF-*κ*B Signaling Pathway *In Vitro*

**DOI:** 10.1155/2022/2108289

**Published:** 2022-03-25

**Authors:** Sheng-Yao Hsu, Shih-Chieh Lee, Hsin-Chung Liu, Shu-Fen Peng, Fu-Shin Chueh, Tai-Jung Lu, Hsu-Tung Lee, Yu-Cheng Chou

**Affiliations:** ^1^Department of Ophthalmology, An Nan Hospital, China Medical University, Tainan, Taiwan; ^2^Department of Optometry, Chung Hwa University of Medical Technology, Tainan, Taiwan; ^3^Department of Food Science and Biotechnology, Da-Yeh University, Dacun, Changhua 515, Taiwan; ^4^Department of Biological Science and Technology, China Medical University, Taichung 404, Taiwan; ^5^Department of Medical Research, China Medical University Hospital, Taichung 404, Taiwan; ^6^Department of Food Nutrition and Health Biotechnology, Asia University, Taichung 413, Taiwan; ^7^Cancer Prevention and Control Center, Taichung Veterans General Hospital, Taichung 407, Taiwan; ^8^Graduate Institute of Medical Sciences, National Defense Medical Center, Taipei 114, Taiwan; ^9^College of Medicine, National Chung Hsing University, Taichung 402, Taiwan; ^10^Department of Neurosurgery, Neurological Institute, Taichung Veterans General Hospital, Taichung 407, Taiwan; ^11^Department of Applied Chemistry, National Chi Nan University, Nantou 545, Taiwan; ^12^Department of Neurological Surgery, Tri-Service General Hospital, National Defense Medical Center, Taipei 114, Taiwan

## Abstract

Phenethyl isothiocyanate (PEITC), extracted from cruciferous vegetables, showed anticancer activity in many human cancer cells. Our previous studies disclosed the anticancer activity of PEITC in human glioblastoma multiforme (GBM) 8401 cells, including suppressing the cell proliferation, inducing apoptotic cell death, and suppressing cell migration and invasion. Furthermore, PEITC also inhibited the growth of xenograft tumors of human glioblastoma cells. We are the first to investigate PEITC effects on the receptor tyrosine kinase (RTK) signaling pathway and the effects of proinflammatory cytokines on glioblastoma. The cell viability was analyzed by flow cytometric assay. The protein levels and mRNA expressions of cytokines, including tumor necrosis factor-*α* (TNF-*α*), interleukin-1*β* (IL-1*β*), and interleukin-6 (IL-6), were determined by enzyme-linked immunosorbent assay (ELISA) reader and real-time polymerase chain reaction (PCR) analysis, respectively. Furthermore, nuclear factor-kappa B- (NF-*κ*B-) associated proteins were evaluated by western blotting. NF-*κ*B expression and nuclear translocation were confirmed by confocal laser microscopy. NF-*κ*B binding to the DNA was examined by electrophoretic mobility shift assay (EMSA). Our results indicated that PEITC decreased the cell viability and inhibited the protein levels and expressions of IL-1*β*, IL-6, and TNF-*α* genes at the transcriptional level in GBM 8401 cells. PEITC inhibited the binding of NF-*κ*B on promoter site of DNA in GBM 8401 cells. PEITC also altered the protein expressions of protein kinase B (Akt), extracellular signal-regulated kinase (ERK), and NF-*κ*B signaling pathways. The inflammatory responses in human glioblastoma cells may be suppressed by PEITC through the phosphoinositide 3-kinase (PI3K)/Akt/NF-*κ*B signaling pathway. Thus, PEITC may have the potential to be an anti-inflammatory agent for human glioblastoma in the future.

## 1. Introduction

The incidence rate of glioblastoma multiforme (GBM) was 2.9 times in the USA (2.48 per 100,000) and as many as that in Taiwan (0.85 per 100,000) [1]. Patients with GBM had the lowest survival rate in the histology of primary malignant brain and CNS tumors: the one-year survival rate was 37.5% in the USA and 50.3% in Taiwan, respectively. According to a hospital-based study from the National Cancer Database in the USA, even GBM patients treated at an academic medical center and the high-volume facility had the median overall survival of 13.3 months [2]. Current multimodality treatments cannot control this most common and aggressive primary brain malignancy well.

The complex pathogenesis in GBM involves receptor tyrosine kinase (RTK) signaling through two main downstream signaling pathways, Ras/mitogen-activated protein kinase (MAPK)/extracellular signal-regulated kinase (ERK) and Ras/phosphoinositide 3-kinase (PI3K)/protein kinase B (Akt) [3]. Besides, inhibition of the ERK/NF-*κ*B signaling pathway can block GBM progression [4]. Cytokines including tumor necrosis factor-*α* (TNF-*α*) and interleukin-1*β* (IL-1*β*), pathogen-associated molecular patterns, ultraviolet and ionizing radiation, reactive oxygen species, growth factors, DNA damage, and oncogenic stress can trigger NF-*κ*B activation pathways [5]. TNF-*α* is a proinflammatory cytokine with pleiotropy and biological effects [6]. However, the Akt pathway triggers critical immune and inflammatory responses in human embryonic kidney 293 cells [7]. It activates NF-*κ*B by tumor necrosis factor (TNF). High levels of inflammatory cytokines such as IL-1*β*, IL-6, and IL-8 enhance cell proliferation, invasion, stemness, and angiogenesis [8]. Furthermore, the elevated inflammatory cytokine IL-6 can raise tumor progression and invasion in GBM, and high levels of IL-1*β* also activate GBM cells and promote IL-6 production [9].

Phenethyl isothiocyanate (PEITC), a component extracted from cruciferous vegetables, exhibits chemopreventive activity in diverse tumors. It has been investigated in small human clinical trials against various diseases from cancer to autism [10]. PEITC targets proteins that inhibit different cancer-promoting mechanisms, including cell proliferation, progression, and metastasis [11]. Our previous studies disclosed the *in vitro* effects of PEITC on human GBM 8401 cells, including the apoptosis induction [12], the reduction of migration and invasion through the inhibition of uPA, Rho A, and Ras, as well as the inhibition of matrix metalloproteinase gene expression [13], and the changes of the gene expressions and the levels of cell cycle regulation-associated proteins [14]. Furthermore, we also revealed that PEITC suppressed the *in vivo* growth of xenograft tumors of human GBM cells [15]. Literature reported that the pretreatment of PEITC promoted the sensitivity of temozolomide- (TMZ-) resistant glioblastoma cell lines and toward TMZ to inhibit the expression of O^6^-methyl-guanine-DNA methyltransferase (MGMT) through suppressing NF-*κ*B activity to reverse the chemoresistance [16].

No reports reveal PEITC effects on RTK signaling pathways and immune-inflammatory responses of GBM in the available literature. In the present study, we first investigated the regulations among ERK, Akt-dependent pathways, NF-*κ*B activity, and cytokine levels in GBM 8401 cells after PEITC treatment *in vitro*.

## 2. Materials and Methods

### 2.1. Chemicals and Reagents

PEITC, Tris-HCl, trypan blue, propidium iodide (PI), and dimethyl sulfoxide (DMSO) were obtained from Sigma Chemical Co. (St. Louis, MO, USA). RPMI-1640, fetal bovine serum (FBS), L-glutamine, penicillin-streptomycin, and trypsin-EDTA were purchased from Gibco BRL/Invitrogen (Carlsbad, CA, USA). IL-1*β* (ab214025), IL-6 (ab178013), and TNF-*α* (ab181421) were purchased from Abcam (Cambridgeshire, UK). Primary antibodies and secondary antibodies were obtained from Cell Signaling Technology (St. Louis, MO, USA). Polyvinylidene difluoride (PVDF) membrane was obtained from Millipore (Temecula, CA, USA). PEITC was dissolved in DMSO.

### 2.2. Cell Culture

Human brain glioblastoma multiforme (GBM) 8401 cell line was purchased from the Food Industry Research and Development Institute (Hsinchu, Taiwan). Cells were cultured in RPMI-1640 medium supplemented with 10% FBS, 2 mM L-glutamine, and 1% antibiotics (100 units/ml penicillin and 100 *μ*g/ml streptomycin), grown at 37°C under a humidified 5% CO_2_ and 95% air at one atmosphere. The medium was changed every two days [17].

### 2.3. Cell Morphological Observation and Cell Viability Measurement

GBM 8401 cells at a density of 1 × 10^5^ cells/well were plated in 12-well plates and were treated with PEITC at the final concentrations (0, 4, 8, and 12 *μ*M) for 48 h. Cells from each well were monitored for morphological examination, and representative photographs were taken at ×200 magnification under an inverted microscope. To determine cell viability, cells from the individual well were trypsinized and collected by centrifuging at 1500 rpm for 5 min, washed twice with PBS, and added PI solution (5 *μ*g/ml). Nonviable cells were stained with PI dye and displayed brighter fluorescence than the viable cells by flow cytometric analysis (FACSCalibur, Becton-Dickinson; San Jose, CA, USA) [18].

### 2.4. IL-1*β*, IL-6, and TNF-*α* Determination by Enzyme-Linked Immunosorbent Assay (ELISA) Reader

The GBM 8401 cells (2.5 × 10^5^ cells) in RPMI-1640 medium containing 10% fetal bovine serum (FBS), 2 mM L-glutamine, 100 units/ml penicillin, and 100 *μ*g/ml streptomycin with various concentrations of PEITC (0, 4, 8, and 12 *μ*M) were placed onto a 24-well culture plate for 48 h. At the end of incubation, cells were centrifuged and medium was collected for ELISA. In brief, 50 *μ*l of medium was added to 50 *μ*l of the antibody cocktail and was incubated for 1 hour at room temperature. Each well was washed with 1x wash buffer, and 100 *μ*l of development solution was added to each well and incubated for 10 minutes in the dark. 100 *μ*l of stop solution was added to well for ELISA Reader, set the OD at 450 nm as described previously [19].

### 2.5. Real-Time Polymerase Chain Reaction (RT-PCR)

GBM 8401 cells (2.4 × 10^6^ cells/dish) were plated to 10 cm dishes overnight and then exposed to 0 and 8 *μ*M of PEITC for 24 h. Cells from the individual sample were collected, and the total RNA was isolated using the Qiagen RNeasy Mini Kit (Qiagen, Inc., Valencia, CA, USA) as described previously [20–22]. RNA samples were reverse-transcribed to cDNA at 42°C for 30 min using the High-Capacity cDNA Reverse Transcription Kit. A defined amount of cDNA was mixed with the Master Mix containing SYBR Green and 200 nM of primers shown in Table 1. Then, quantitative PCR was performed by 50°C for 2 min, 95°C for 10 min, and 40 cycles of 95°C for 15 sec and 60°C for 1 min using the Applied Biosystems 7300 Real-Time PCR System in triplicate. The fold change of gene expression was determined using the comparative 2^-*ΔΔ*CT^ method based on comparing with the level of GAPDH.

### 2.6. Western Blotting Assay

GBM 8401 cells (1 × 10^6^ cells/dish) were plated in 10 cm dishes and treated with 0 and 8 *μ*M of PEITC for 0, 6, 24, and 48 h. After treatment, cells were collected and lysed in lysate buffer composed of 40 mM Tris-HCl (pH 7.4), 10 mM EDTA, 120 mM NaCl, 1 mM dithiothreitol, and 0.1% Nonide P-40. The protein concentration of each treatment was determined by using the Bio-Rad protein assay kit. Defined amounts (30 *μ*g) of proteins from individual samples were separated on 10% sodium dodecyl sulfate-polyacrylamide electrophoretic gels (SDS-PAGE) and then electrotransferred to PVDF membranes (Millipore, Temecula, CA, USA). The resultant blot was soaked in blocking buffer, 2.5% FBS in TBST (Tris-buffered saline containing Tween-20) for 1 h at room temperature. Then, the blots were probed with the primary antibodies for t-ERK1/2, p-ERK1/2^Thr202/Tyr204^, PI3K, p-Akt1/PKB*α*^Thr308^, p-Akt1/PKB*α*^Ser473^, Akt, p-p65^Ser276^, p-p65^Ser529^, p65, p-IKK*α*/*β*^Thr23^, IKK*α*/*β*, p-I*κ*B*α*^Ser32/Ser36^, and *β*-actin (Cell Signaling Technology; Beverly, MA, USA) in blocking buffer at 4°C overnight. Immunoreactive proteins were reacted with horseradish peroxidase- (HRP-) conjugated secondary antibodies (Cell Signaling Technology; Beverly, MA, USA) and detected by chemiluminescence. The relative protein expression from each treatment was assessed by ImageJ software as described previously [23].

### 2.7. Observations of Confocal Laser Scanning Microscopy

GBM 8401 cells at a density of 1 × 10^5^ cells/well were maintained on 18 mm coverslips and then treated with PEITC (0 and 8 *μ*M) for 24 h. At the end of treatment, cells were fixed with 4% paraformaldehyde in PBS and permeabilized using 0.2% Triton-X 100 in PBS for 15 min. Subsequently, cells were washed with PBS and probed with an anti-p65 antibody (Novus Biologicals; Centennial, CO, USA) and then reacted with secondary antibodies conjugated with FITC (green fluorescence), and their nucleus was stained by PI (red fluorescence). All samples were observed and photographed under a Leica TCS SP8 Confocal Spectral Microscope, as described previously [24].

### 2.8. Electrophoretic Mobility Shift Assay (EMSA)

GBM 8401 cells (5 × 10^5^ cells/dish) were plated into 10 cm dishes, and were incubated with 0, 4, 8, and 12 *μ*M of PEITC for 24 h. Cells were harvested for nuclear extracts by using the NE-PER Nuclear and Cytoplasmic Extraction Kit (Pierce, Rockford, Illinois, USA), and the protein concentrations for EMSA were determined with a LightShift Chemiluminescent EMSA Kit (Pierce) as described previously [22].

### 2.9. Statistical Analysis

All data were represented with the mean ± standard error from at least three independent experiments. One-way analysis of variance (ANOVA) with Newman-Keuls multicomparison test was used for the comparison between PEITC-treated and control groups. The difference between PEITC-treated and control was considered significant if *p* < 0.05.

## 3. Results

### 3.1. PEITC Decreased the Cell Viability of GBM 8401 Cells

**Figure 1:**
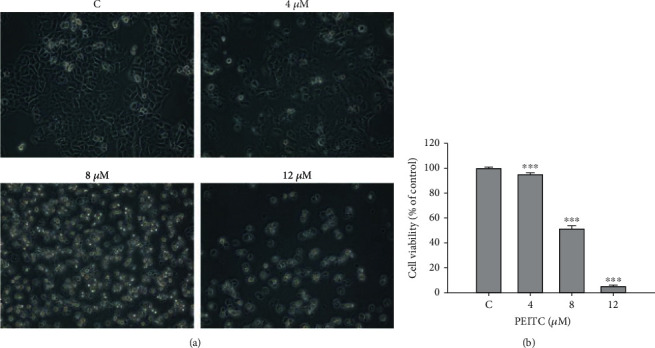
PEITC induced cell morphological changes and decreased the viable cell number of GBM 8401 cells. (a) Cells were treated with defined concentrations (0, 4, 8, and 12 *μ*M) of PEITC for 48 h, and cell morphological alternations were monitored under a phase-contrast microscope at ×200 as described in Materials and Methods. (b) Cells were harvested to determine the viable cell number by flow cytometric assay. The values presented are the mean ± SD (*n* = 3) from three independent experiments. ^∗∗∗^*p* < 0.001, significant difference compared for PEITC-treated and vehicle control cells. C: control.

GBM 8401 cells were treated with PEITC at different concentrations (0, 4, 8, and 12 *μ*M) for 48 h before the cells were analyzed. The cell morphology was monitored, and the cytotoxicity of PEITC treatment was determined. PEITC induced morphological alternations of GBM 8401 cells based on cells that became smaller in size, shrinking, membrane blebbing, and floated on medium (Figure 1(a)). The total percentages of viable cells were analyzed by PI exclusion assay using flow cytometric assay, and results showed that PEITC diminished the number of viable GBM 8401 cells dose dependently (Figure 1(b)). After being exposed to more than 4 *μ*M of PEITC, the total viable cells were significantly reduced in GBM 8401 cells. PEITC at 8 *μ*M reduced cell viability to 52.4% in GBM 8401 cells, and more than 90% reduction of cells exposed to 12 *μ*M of PEITC was observed after 48 h treatment. Thus, 8 *μ*M of PEITC was selected for subsequent experiments.

### 3.2. PEITC Inhibited the Levels and mRNA Transcription of IL-1*β*, IL-6, and TNF-*α* Genes in GBM 8401 Cells

**Figure 2:**
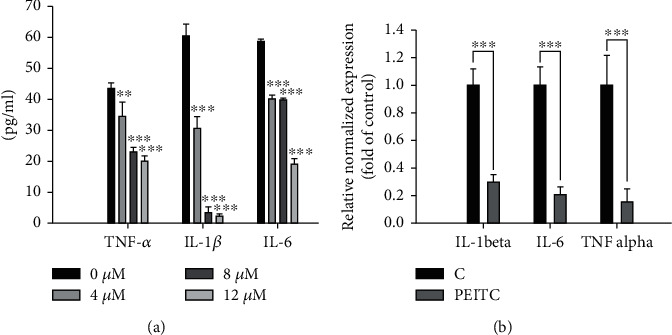
PEITC inhibited the mRNA levels of cytokines in GBM 8401 cells. Cells were placed in 12-well plates and treated with 0, 4, 8, and 12 *μ*M of PEITC for 24 h. Samples were assayed for the proteins levels of IL-1*β*, IL-6, and TNF-*α* by ELISA (a). Or cells were treated with 0 and 8 *μ*M of PEITC for 24 h. Individual RNA samples were isolated and then reverse-transcribed to obtain cDNA for real-time PCR as described in Materials and Methods. The expression of IL-1*β*, IL-6, and TNF-*α* genes was normalized by comparing them with that of GAPDH (b). Data represent the mean ± SD of three experiments. ^∗∗^*p* < 0.01 and ^∗∗∗^*p* < 0.001, significantly different between the PEITC-treated and control groups.

The effects of PEITC on the levels (proteins) and mRNA transcription of cytokine genes, including IL-1*β*, IL-6, and TNF-*α*, were investigated by ELISA reader and real-time PCR analysis, respectively (Figures 2(a) and 2(b)). The levels (proteins) of IL-1*β*, IL-6, and TNF-*α* on GBM 8401 cells were of significant inhibition, and these effects were dose dependent (Figure 2(a)). Moreover, the mRNA expressions of IL-1*β*, IL-6, and TNF-*α* were indeed reduced 70%, 79.1%, and 84.5%, respectively, when GBM 8401 cells were exposed to 8 *μ*M of PEITC for 24 h (^∗∗∗^*p* < 0.001) compared to the control group (Figure 2(b)). Our data suggested that PEITC might regulate the expressions of IL-1*β*, IL-6, and TNF-*α* at the transcriptional level in GBM 8401 cells.

### 3.3. PEITC Altered Akt- and ERK-Associated Protein Expression in GBM 8401 Cells

**Figure 3:**
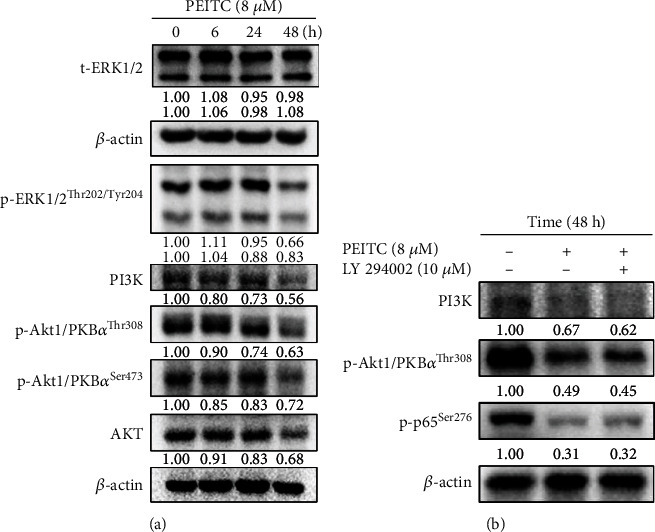
PEITC affected Akt- and ERK-associated proteins in GBM 8401 cells. (a) Cells were exposed to 0 and 8 *μ*M of PEITC for 0, 6, 24, and 48 h and then harvested to measure the levels of Akt- and ERK-associated proteins, including t-ERK1/2, p-ERK1/2^Thr202/Tyr204^, PI3K, p-Akt1/PKB*α*^Thr308^, p-Akt1/PKB*α*^Ser473^, and Akt in GBM 8401 cells or (b) cells were pretreated with PI3K inhibitor (LY 294002) and were collected for western blotting assay as described in Materials and Methods.

MAPK and Akt signaling pathways involved in the secretion of TNF-*α* cytokine in GBM 8401 cells were investigated in this study. By western blotting analysis, PEITC at 8 *μ*M decreased the protein levels of p-ERK1/2^Thr202/Tyr204^ at 24 and 48 h treatment time dependently but did not change the protein levels of t-ERK1/2 significantly at 6, 24, and 48 h treatment (Figure 3(a)). Moreover, PEITC at 8 *μ*M reduced the protein levels of PI3K, p-Akt1/PKB*α*^Thr308^, p-Akt1/PKB*α*^Ser473^, and Akt at 6, 24, and 48 h treatment in a time-dependent manner, respectively (Figure 3(b)). We also investigated the effects of PI3K inhibitor (LY 294002) pretreatment on GBM 8401 cells, and then, GBM 8401 cells were treated with PEITC for 48 h. Cells were harvested for western blotting for the expressions of PI3K, p-Akt1/PKB*α*^Thr308^, and p-p65^Ser276^ in GBM 8401 cells (Figure 3(b)). Both cotreatments of PEITC and LY 94002 resulted in lower PI3K and PKB*α*^Thr308^ in GBM 8401 cells; however, there is no significant change in the levels of p-p65^Ser276^.

### 3.4. PEITC Altered NF-*κ*B Signaling Pathway-Associated Protein Levels, NF-*κ*B Translocation, and NF-*κ*B Activity in GBM 8401 Cells

**Figure 4:**
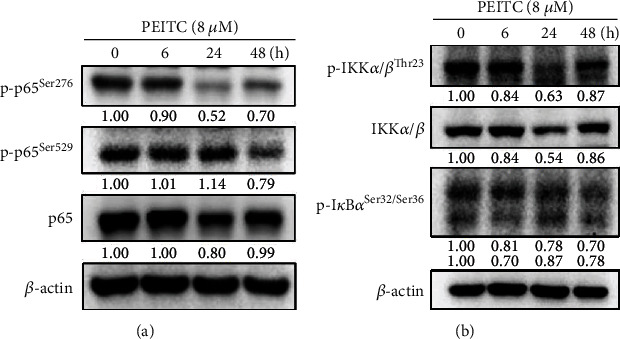
PEITC affected NF-*κ*B-associated proteins in GBM 8401 cells. Cells were exposed to 0 and 8 *μ*M of PEITC for 0, 6, 24, and 48 h and then harvested to determine the levels of proteins related to NF-*κ*B-associated signaling pathways in GBM 8401 cells by western blotting assay as described in Materials and Methods: (a) p-p65^Ser276^, p-p65^Ser529^, and p65; (b) p-IKK*α*/*β*^Thr23^, IKK*α*/*β*, and p-I*κ*B*α*^Ser32/Ser36^.

**Figure 5:**
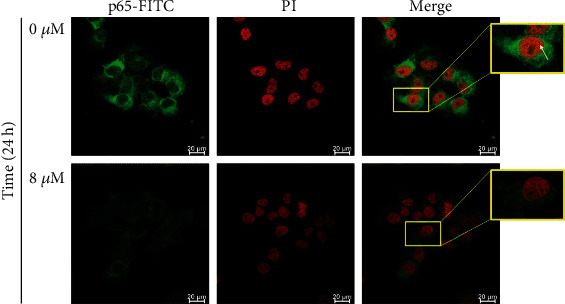
PEITC affected NF-*κ*B expression and nuclear translocation in GBM 8401 cells. Cells were treated with 0 and 8 *μ*M of PEITC for 24 h, and then, the expression and nuclear translocation of NF-*κ*B (p65) in GBM 8401 cells were observed by confocal laser scanning microscopy as described in Materials and Methods.

The effects of PEITC on the TNF-*α* cytokine secretion were investigated for the involvement of the NF-*κ*B signaling pathway. By western blotting analysis, PEITC at 8 *μ*M decreased the protein levels of NF-*κ*B (p-p65^Ser276^) at 6, 24, and 48 h treatment and NF-*κ*B (p-p65^Ser529^) at 48 h (Figure 4(a)). PEITC at 8 *μ*M decreased the protein levels of NF-*κ*B (p65) at 24 and 48 h treatment in a time-dependent manner (Figure 4(a)). PEITC also reduced the protein levels of p-IKK*α*/*β*^Thr23^, IKK*α*/*β*, and p-I*κ*B*α*^Ser32/Ser36^ by western blotting analysis in time-dependent manners (Figure 4(b)). Furthermore, PEITC at 8 *μ*M abated the expression and nuclear translocation of NF-*κ*B (p65) in GBM 8401 cells at 24 h, which were observed by confocal laser scanning microscopy (Figure 5).

### 3.5. PEITC Decreased the Binding of NF-*κ*B p65 on DNA in GBM 8401 Cells

**Figure 6:**
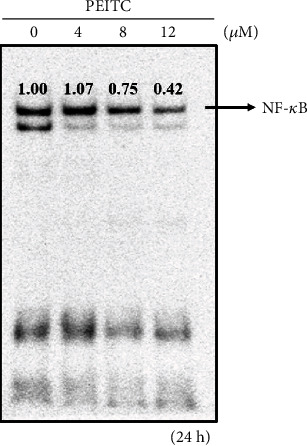
PEITC decreased the binding of NF-*κ*B p65 on DNA in GBM 8401 cells. GBM 8401 cells (5 × 10^5^ cells) were treated with 0, 4, 8, and 12 *μ*M of PEITC for 24 h. Cells were harvested for nuclear extracts, and the protein concentrations for EMSA were determined with a LightShift Chemiluminescent EMSA Kit (Pierce) as described in Materials and Methods.

In order to further confirm the effects of PEITC on NF-*κ*B p65 binding on DNA in GBM 8401 cells, cells were incubated with various concentrations of PEITC (0, 4, 8, and 12 *μ*M) for 24 h and were collected and further assayed by using EMSA and results are shown in Figure 6. Results from Figure 6 show that NF-*κ*B p65 binding on nuclear DNA was decreased at 25% and 58% at 8 and 12 *μ*M of PEITC treatment, respectively.

## 4. Discussion

PEITC prevents the initiation of carcinogenesis and suppresses the progression of tumorigenesis [11]. The anticancer effects of PEITC on cell proliferation, apoptosis, angiogenesis, metastasis, autophagy, inflammation, and immunomodulation in different cancer models have been reported. PEITC reduced the cell viability of GBM 8401 cells in our previous experiments, including the studies of apoptosis, migration, and invasion [12, 13]. In the present study, PEITC changed the morphology of GBM 8401 cells (Figure 1(a)). PEITC reduced cell viability of GBM 8401 cells after 48 h treatment in a dose-dependent manner (Figure 1(b)), and the viability was decreased to 52.4% at 8 *μ*M of PEITC treatment.

It is well documented that cytokines such as IL-1*β*, IL-6, and TNF-*α* were involved in inflammatory responses after host was exposed to environmental antigen. However, the excessive release of those inflammatory mediators may result in chronic inflammatory diseases if they are out of control. Thus, IL-1*β* or IL-6, TNF-*α* may be a target to control the inflammatory responses. Moreover, IL-1*β* and/or TNF-*α* have been shown to induce the expression of IL-6 in various tissues and cell types [25–29].

Therefore, we investigated whether or not PEITC affected the levels (protein) of IL-1*β*, IL-6, and TNF-*α* in GBM 8401 cells after treatment with or without PEITC at 0, 4, 8, and 12 *μ*M for 24 h and were assayed by an ELISA reader. The results (Figure 2(a)) indicated that PEITC at 8 and 12 *μ*M significantly inhibited the levels of IL-1*β*, IL-6, and TNF-*α* and higher concentrations of PEITC lead to higher inhibitions. The gene expression of IL-1*β*, IL-6, and TNF-*α* was inhibited by PEITC in a similar trend in GBM 8401 cells (Figure 2(b)).

RTK signaling regulates cell proliferation, survival, metastasis, and angiogenesis in GBM cells through the Ras/MAPK/ERK and Ras/PI3K/AKT pathway, two main downstream of RTK [3]. PEITC plays multiple biological functions in human cancer cells. PEITC inhibited the invasion and migration of human colon cancer HT29 cells by decreasing SOS-1, PKC, ERK1/2, and Rho A which led to the reduction of MMP-2 and MMP-9. PEITC also interfered with the expressions of Ras, FAK, and PI3K and suppressed GRB2, NF-*κ*B, iNOS, and COX-2, which resulted in inhibiting cell proliferation in HT29 cells [30]. In the human leukemia xenograft animal model, PEITC induced tumor cell apoptosis and reduced tumor growth via downregulations of AKT, JNK, and Mcl-1 [31]. PEITC repressed protein and gene expressions concerning Toll-like receptor 3- (TLR3-) mediated IFN regulatory factor 3 (IRF3) signaling pathway *in vitro* and *in vivo* [32]. TLR3 upon dsRNA binding involves its specific adaptor Toll/IL-1R domain-containing adapter protein inducing IFN-*β* to enhance the signal resulting in NF-*κ*B- or IRF3-mediated upregulation of proinflammatory and cytokine genes. PEITC also diminished the phosphorylation of epidermal growth factor receptor (EGFR), PI3K (p85), 3-phosphoinositide-dependent protein kinase 1 (PDK1), Akt, phosphorylated IKK, and I*κ*B to inactivate NF-*κ*B in human oral squamous carcinoma cells (SAS cells) [33]. Besides, PEITC launches the MAPK signaling pathway through the elevated expression of phosphorylated p38, JNK, and ERK. In our study, PEITC decreased the protein levels of p-ERK1/2^Thr202/Tyr204^, PI3K, p-Akt1/PKB*α*^Thr308^, p-Akt1/PKB*α*^Ser473^, and Akt in time-dependent manners (Figure 3(a)). Both cotreatments of PEITC and LY 94002 decreased PI3K and p-Akt/PKB*α*^Thr308^ in GBM 8401 cells (Figure 3(b)). PEITC changed the levels of Akt- and ERK-associated proteins in GBM 8401 cells and may modulate several critical cellular pathways involving cell proliferation, survival, migration, and angiogenesis.

NF-*κ*B is involved in the early phases of the cell cycle and regulates cell growth, differentiation, immune, and inflammatory responses [34]. Activation of NF-*κ*B enhances the initiation and progression of tumors through the mechanism of angiogenesis, metastasis, and reprogramming of metabolism [5]. A heterodimer of the p50 and p65 subunits is the most widely studied form of NF-*κ*B. NF-*κ*B in the cytoplasm is bound in an inactive complex with I*κ*B, a natural biological inhibitor of NF-*κ*B, in most cells [35]. I*κ*B*α*, I*κ*B*β*, p105/I*κ*B*γ* (precursor of p50), p100 (precursor of p52), and I*κ*B*ε* belong to the I*κ*B family [36]. IkB kinase complex results in the phosphorylation of I*κ*B*α* at serines 32 and 36 or I*κ*B*β* at serines 19 and 23 [37]. The phosphorylation of I*κ*B*α* and I*κ*B*β* targeted I*κ*B for ubiquitin-dependent degradation through the 26S proteasome complex and resulted in the release and nuclear translocation of NF-*κ*B [38]. NF-*κ*B is highly active in glioblastoma, promoting cell aggressiveness [39] and inflammatory niche [40]. NF-*κ*B activity was also associated with shorter survival in glioma patients [41]. Targeting the NF-*κ*B-FAT1 axis might inhibit the important tumor-promoting pathway in glioblastoma because FAT1 and NF-*κ*B independently enhance protumorigenic inflammation and upregulate the expression of HIF-1*α*/EMT/stemness in tumors [42]. PEITC revoked receptor activator of NF-*κ*B ligand- (RANKL-) induced degradation of I*κ*B-*α*, a suppressive partner of NF-*κ*B in RAW264.7 macrophages, and prohibited the activation of ERK1/2 and p38 MAPK from decreasing RANKL-induced osteoclastogenesis [43].

The NF-*κ*B signaling pathway plays a critical role in anticancer mechanism. Cellular migration and invasion, which were induced by DLL4, could be inhibited by either *β*-catenin or a p50 inhibitor in glioblastoma U87MG and U251 cells [44]. The migration and invasion of glioma cells are synergistically promoted by Notch activation-stimulated *β*-catenin and NF-*κ*B signaling pathways. The suppression of NF-*κ*B binding activity may implicate in the inhibition of MMP in GBM 8401 cells, and several critical metastasis-related proteins, such as p-EGFR^Tyr1068^, SOS-1, GRB2, Ras, p-AKT^Ser473^ and p-AKT^Thr308^, NF-*κ*B-p65, Snail, E-cadherin, N-cadherin, NF-*κ*B, MMP-2, and MMP-9, were decreased by tetrandrine from our previous study [45]. In this study, PEITC reduced the protein levels of p-ERK1/2^Thr202/Tyr204^, PI3K, p-Akt1/PKB*α*^Thr308^, p-Akt1/PKB*α*^Ser473^, and Akt in time-dependent manners by western blotting analysis (Figure 3(a)). PEITC at 8 *μ*M decreased the levels of NF-*κ*B (p-p65^Ser276^, p-p65^Ser529^, and p65) in a time-dependent manner by western blotting analysis (Figure 4(a)). PEITC diminished the levels of p-IKK*α*/*β*^Thr23^, IKK*α*/*β*, and p-I*κ*B*α*^Ser32/Ser36^ after 6, 24, and 48 h treatment (Figure 4(b)). PEITC at 8 *μ*M also abated the expression and nuclear translocation of NF-*κ*B (p65) in GBM 8401 cells at 48 h by confocal laser scanning microscopy (Figure 5). These results indicated that PEITC affected the NF-*κ*B signaling pathway and may affect the aggressiveness of glioblastoma and the inflammatory microenvironment. In our previous study, demethoxycurcumin inhibited the motility, migration, and invasion of GBM 8401 cells via inhibition of PI3K/Akt and NF-*κ*B signaling pathways [46]. PEITC reduced migration and invasion through the inhibition of uPA, Rho A, and Ras with inhibition of matrix metalloproteinase gene expression in GBM 8401 cells [13]. Taken together, PEITC may also suppress the migration and invasion of GBM 8401 cells through Akt, ERK, and NF-*κ*B signaling pathways.

Furthermore, PEITC reversed the TMZ resistance of glioblastoma cells (U373-R, U87-R, and T98G cells) by suppressing MGMT via inhibiting the NF-*κ*B activity [16]. Inhibition of the NF-*κ*B activity increased the sensitivity of glioblastoma cells to alkylating agents such as TMZ in patients with acquired or induced chemoresistance. PEITC also inhibited cell growth in the U373-R grafted xenograft mouse model. In our study in A375.S2 human melanoma cancer cells *in vitro*, PEITC suppressed cell migration and invasion by affecting the MAPK signaling pathway [47]. p-AKT^Ser473^ levels were increased by PEITC at 1-2.5 *μ*M at 24 h, but decreased at 48 h treatment. PEITC at 2.5 *μ*M decreased NF-*κ*B binding of p65 to DNA in A375.S2 cells, but at 1-2 *μ*M, it increased the binding. In the present study, PEITC at 8 *μ*M decreased the protein levels of PI3K, p-Akt1/PKB*α*^Thr308^, p-Akt1/PKB*α*^Ser473^, and Akt at 6, 24, and 48 h treatment in a time-dependent manner in GBM 8401 cells, respectively (Figure 3(a)). PEITC at 8 *μ*M decreased the protein levels of NF-*κ*B (p-p65^Ser276^) at 6, 24, and 48 h treatment in a time-dependent manner, and NF-*κ*B (p-p65^Ser529^) at 48 h treatment, respectively (Figure 4(a)). PEITC at 8 *μ*M decreased the protein levels of NF-*κ*B (p65) at 24 and 48 h treatment in a time-dependent manner (Figure 4(a)). PEITC may have different effects on MAPK and NF-*κ*B signaling pathways in the same cancer cells at different concentrations and treatment timing. Furthermore, results from EMSA indicated that PEITC at 4 and 8 *μ*M significantly inhibited the binding of NF-*κ*B p65 on DNA in GBM 8401 cells (Figure 6). Therefore, further studies of the directions of these signaling pathways in glioblastoma cells at different concentrations and treatment timing of PEITC are needed.

IL-1*β*, a major proinflammatory cytokine, launches various malignant processes by activating different cells to increase key molecules driving oncogenic events [8]. A high level of IL-1*β* was observed in glioblastoma cells (CCF3 and U87MG cells) [48] and human glioblastoma specimens [49]. The binding of IL-1*β* and the IL-1R leads to activating NF-*κ*B and MAPK signaling pathways and cooperatively induces the expression of target genes cooperatively [50]. IL-1*β*-dependent activation of NF-*κ*B, p38 MAPK, and JNKs pathways, however, increases VEGF and sphingosine kinase 1, subsequently enhancing migration, invasion, and angiogenesis, respectively [8, 51]. GBM cells regain self-renewal capacity after exposure to IL-1*β* [52]. Furthermore, IL-1*β* and TGF-*β* cooperated to elicit upregulation of stemness factor genes and augmented invasiveness and drug resistance, leading to tumor growth *in vivo* [53]. Therefore, targeting the production and activity of IL-1*β* might control the progression of glioblastoma.

The level of IL-6 mRNA was stabilized, and IL-6 biosynthesis was increased by the activation of several signaling pathways by proinflammatory cytokines IL-1*β* or TNF-*α* [54]. IL-6-mediated STAT3 activation enhanced cell migration and invasion in glioblastoma cells (U251, T98G, and U87MG) [55]. TNF and the associated receptor superfamily are important to the development of glioblastoma, and upregulation of TNF-*α* is influential to the progression of glioblastoma in U373 glioma cells [56]. Targeting TNF superfamily-related genes may be a potential therapeutic approach for GBM [57]. In our study, PEITC inhibited the transcription of IL-1*β*, IL-6, and TNF-*α* genes in GBM 8401 cells (Figure 2(b)) and may control the progression of GBM through targeting IL-1*β* or affecting IL-6 on the regulation of signaling pathways by proinflammatory cytokines IL-1*β* or TNF-*α*. The detailed mechanism needs to be confirmed in *in vivo* studies in the future.

## 5. Conclusions

**Figure 7:**
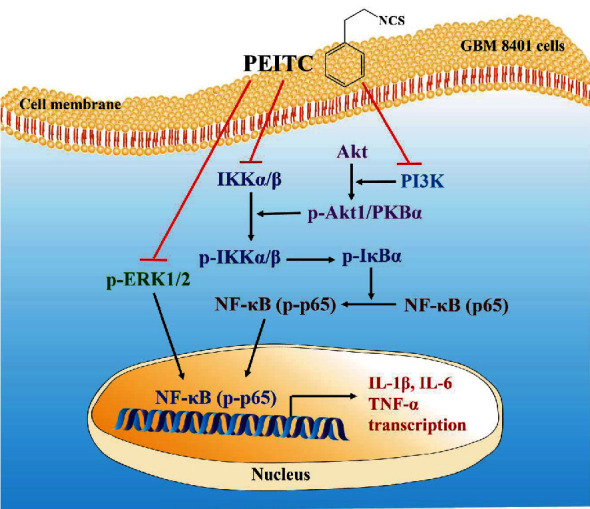
The possible signaling pathways involved in suppressing proinflammatory cytokines in human glioblastoma cells by PEITC.

PEITC significantly reduced the levels of proinflammatory cytokines, such as TNF-*α*, IL-6, and IL-1*β* genes, in transcriptional levels and modulated ERK- and Akt-dependent and NF-*κ*B signaling pathways in GBM 8401 cells. The possible signaling pathways regarding PEITC on GBM 8401 cells are summarized (Figure 7). PEITC may have anti-inflammatory effects on GBM, which can be a basis for further experiments to explore the immune regulation of PEITC on glioblastoma *in vivo*.

## Figures and Tables

**Table 1:** Primer sequences used for real-time PCR.

Primer name	Primer sequence	
TNF-*α*	F	5′-ATTGCCCTGT GAGGAGGAC-3′
	R	5′-TGAGCCAGAAGAGGTTG AGG-3′
IL-1*β*	F	5′-GGA TATGGAGCAACAAGTGG-3′
	R	5′-ATGTACCAG TTGGGGAACTG-3′
IL-6	F	5′-CTTCGGTCCAGTTGCCTTCT-3′
	R	5′-GTGAGTGGCTGTCTGTGTGG-3′
GAPDH	F	5′-TGCACCACCAACTGCTTAGC-3′
	R	5′-GGCAT GGACTGTGGTCATGAG-3′

Abbreviations: GAPDH: glyceraldehyde-3-phosphate dehydrogenase; F: forward primers; R: reverse primers.

## Data Availability

The datasets applied and analyzed in the present study are available from the corresponding author on reasonable request.
